# Effects of Deep Cervical Flexor Training on Forward Head Posture, Neck Pain, and Functional Status in Adolescents Using Computer Regularly

**DOI:** 10.1155/2020/8327565

**Published:** 2020-10-05

**Authors:** Isha Sikka, Chandan Chawla, Shveta Seth, Ahmad H. Alghadir, Masood Khan

**Affiliations:** ^1^Optum, Noida 201304, India; ^2^Ability Physiotherapy and Sports Injury Clinic, Hauz Khas, New Delhi 110016, India; ^3^AIIMS, New Delhi 110029, India; ^4^Rehabilitation Research Chair, College of Applied Medical Sciences, King Saud University, P.O. Box 10219, Riyadh 11433, Saudi Arabia

## Abstract

In contemporary societies, computer use by children is a necessity and thus highly prevalent. Using computers for long hours is related to a higher risk of computer-related muscular disorders like forward head posture (FHP) and neck pain (NP). Deep cervical flexor (DCF) muscles are important head-on-neck posture stabilizers; thus, their training may lead to an improvement in FHP and NP. The aim of this study was to determine if 4 weeks of DCF training is effective in alleviating NP, improving FHP, and functional status in adolescent children using computers regularly, a pretest-posttest experimental group design was used. Subjects were randomly assigned into the experimental group (receiving DCF training and postural education) and the control group (receiving postural education only). 30 subjects with a mean age of 15.7 ± 1.725 years with NP and FHP using computers regularly participated in the study. Dependent variables were measured on day 1 (at baseline) and after 4 weeks of training. Photographic analysis was used for measuring FHP, visual analog scale for NP intensity, and neck disability index for functional status. Data analysis showed that in both groups, no significant improvement occurred in FHP. In both groups, there was a significant improvement in functional status and NP. There was no significant difference between both groups for FHP and NP. There was a significant improvement in functional status in the experimental group in comparison to the control group. Four weeks of DCF training does not cause a significant improvement in FHP in 13 to 18 years old adolescent children using computers regularly.

## 1. Introduction

There is a high prevalence of musculoskeletal-related pain among young people. One of the studies found neck pain to be most common among all musculoskeletal pain syndromes, affecting around 17.2% of adolescents [1]. In comparison to asymptomatic controls, adult patients having neck pain are found to have increased forward head posture (FHP) and impaired performance of neck flexor and extensor muscles [2–4]. It is proposed that more use of information and communication technologies has concurrently increased the prevalence of neck pain. Computer use has been associated with adolescent neck pain, with daily use of computers exceeding 2-3 hours as a threshold for neck pain [5, 6]. Using a computer for long periods results in static posture maintained for a longer time particularly in neck and shoulder regions [7]. Several studies found a relationship between neck pain and computer usage [6, 8, 9]. It has been proposed that the repetitive use of mobile phones, laptops, computers, TVs, video games, and even backpacks has forced the body to adapt to the FHP and kyphosis [10]. One of the studies reported that subjects assumed significant FHP while viewing mobile phones in comparison to standing neutrally. Greater head tilt angle and smaller neck tilt angle were found when subjects looked at mobile phones in comparison to neutral standing posture [11].

The craniovertebral angle (CVA) is defined as an angle made by the intersection of a line joining the midpoint of the tragus of ear to the skin overlying the C7 spinous process and a horizontal line passing through the C7 spinous process. There is a correlation between FHP, neck pain, and CVA. One of the studies reported that subjects having smaller CVA had FHP and were prone to have increased severity of neck pain [12]. There could be both lower cervical flexion and upper cervical extension in FHP [13] and may include tightness of posterior region muscles and weakness and lengthening of anterior cervical muscles [14]. Deep cervical flexor (DCF) muscles have been found to have a significant role in supporting and strengthening of the cervical spine [15]. Studies also suggested that in the case of cervical disorders, a rehabilitation approach will be more effective if DCF muscles are used properly before strengthening of global cervical muscles [13]. The use of a pressure biofeedback unit is also suggested as a more effective way of DCF strengthening than conventional exercises [16–18].

Sitting in flexed static posture is of greater significance especially during adolescence because growth in spinal structures is rapid during this period. Understanding the mechanism of neck pain associated with FHP and its associated changes in the adolescent age group will help us in framing better therapeutic strategies for this particular age group. Also, this would contribute to the understanding of neck pain in older age groups and will raise awareness for the need for early interventions to prevent such problems.

The DCF has an important role in head-on-neck posture stabilization. Chiu et al. reported that DCF training was more effective in suppressing an increase in the severity of neck pain than those patients who did not do DCF training [19]. Another study reported that a rehabilitation program that included DCF training effectively alleviated symptoms of headaches in patients. The best method to specifically activate DCFs and reduce the involvement of SCM muscles is the craniocervical flexion training (CCFT). Since postural abnormalities cause pain and injuries, postural correction and education have been used for the treatment of such pains [20]. As the mechanism of pain development in computer users has been hypothesized to be prolonged duration of holding a static posture, the importance of postural awareness factors in school children has been emphasized. The objective of the present study is to determine if FHP and neck pain are improved with 4 weeks of DCF training in adolescents of 13 to 18 years of age, who use computers regularly. Hence, we hypothesize that 4 weeks of DCF training will significantly improve FHP, functional status, and neck pain in the computer-using adolescents of 13 to 18 years of age.

## 2. Materials and Methods

### 2.1. Study Design

The pretest-posttest experimental design was used. DCF training and postural education were independent variables, and FHP, functional status, and neck pain were dependent variables. FHP was measured through a CVA in photographic analysis, functional status was measured through the neck disability index (NDI) score, and neck pain was measured through the visual analog scale (VAS) score.

### 2.2. Participants

For experimental research, group sizes of about 30 participants are considered minimum size to make a valid generalization [21, 22]. Therefore, 30 subjects, students of 8^th^ to 12^th^ standard from 2 CBSE affiliated schools (16 males and 14 females with a mean age of 15.7 yrs.±1.725), took part in the study (Table 1). Subjects having neck pain with or without headache, of duration more than 3 months and less than 1 year and 6 months, as identified by the body discomfort chart and NDI value less than 24 (mild to moderate disability scores on NDI) were included in the study. Subjects also had to have FHP as identified by a straight line down from the external meatus falling anterior to shoulder and the mid thorax. Subjects were required to use a computer for at least 3 hours a day for at least 4 days a week or more. Subjects with an ongoing or previous history of spinal fracture, history of cervical spinal surgery, neurological signs, inflammatory disease, spinal instability, spinal tumor, spinal infection, spinal cord compression, congenital, or acquired postural deformity were excluded from the study. The study conforms to “The Code of Ethics of the World Medical Association (Declaration of Helsinki).” The ethical committee of the institutional review board (file id: RRC-2019-08) approved this study. This study has been registered in clinicaltrial.gov (ID: NCT04463199). Subjects were selected depending upon inclusion and exclusion criteria, then randomly allocated into either of the 2 groups using lottery with 15 subjects in each group: experimental group and control group. The participants and the researcher were unaware of the random sequence. The outcome assessor was kept blinded to the allocation. The experimental group was given DCF training and postural education however, the control group was given postural education only.

### 2.3. Instrumentation

- The pressure biofeedback unit (Stabilizer TM, Chattanooga Group, INC., Chattanooga, TN)
- Digital camera (Nikon Coolpix L16)
- Image tool UTHSCSA version 3.0 University of Texas Health Sciences Center, San Antonio, TX
- Adjustable camera stand
- VAS and NDI scales
- Plumb line
- Anatomical markers

### 2.4. Protocol

Consent was obtained from the principals of school, parents, and students. Risks and benefits of the study were discussed with them. The study was conducted on school premises. The study was divided into 3 phases: (1) preintervention evaluation, (2) intervention, and (3) postintervention evaluation.

1. Preintervention evaluation: values of dependent variables were taken on day 1 of the study. Subjects were given a body discomfort chart to mark the area of pain/discomfort. NDI and VAS were given to indicate the level of functional status and level of pain, respectively. FHP was measured by calculating the CVA using a digital photography technique. Values taken on day 1 were designated as CVA Pre, NDI Pre, and VAS Pre
2. Intervention: for the experimental group, DCF training and postural education were given for 4 weeks. For the control group, only postural education was given, verbally as well as in print, for 4 weeks. Under the supervision of a physiotherapist, an exercise regimen was performed for a total of 4 weeks
3. Postintervention evaluation: CVA, NDI, and VAS were again measured at the end of 4^th^ week and designated as CVA Post, NDI Post, and VAS Post

### 2.5. Measurement of FHP

FHP was measured by taking lateral photographs, and then, these photographs were analyzed through a digital photography technique with the help of digitizing software (Image tool UTHSCSA version 3.0). The CVA was measured by software by drawing a line from the tragus of the ear to the 7^th^ cervical vertebrae. The angle this line makes with horizontal is the CVA. The subjects sat on a chair. A plumb line fixed to the wall served as a reference for verticality and was included in the picture. The digital camera was mounted on an adjustable tripod camera stand. At a distance of 0.8 m from the subject, the camera was placed at the level of the subject's head and neck region. The camera base was adjusted to the subject's shoulder height. The subjects were asked to look directly ahead. C7 spinous process was palpated; C7 spinous process and tragus of the ear were marked. A retroreflective marker was placed over the skin at the level of the C7 spinous process and secured with tape. A total of 3 lateral photographs of the subjects were taken, and an average of the CVA was recorded as the final score. The markers were highlighted in the photographs and analyzed using computer software.

### 2.6. Intervention

#### 2.6.1. Craniocervical Flexion Training

*(1) Preparation of Subjects*. Subjects were made to lie in crook lying position with their craniocervical region in midrange neutral position. Folded towels of appropriate thickness were placed under the head not the neck, if required, to maintain the cervical spine's neutral position.

*(2) Preparation of a Pressure Biofeedback Unit (PBU)*. PBU airbag was clipped together and folded in, fastened, and placed suboccipital. The uninflated pressure sensor was kept below the neck so that it touched the occiput then inflated to a stable baseline pressure of 20 mmHg to just fill the space below the neck but not to push it into lordosis.

*(3) Patient Instruction*. Subjects were demonstrated the correct action of the DCF that were gentle nodding of head as if saying “yes.” With gentle nodding, the patient was instructed to target one mark corresponding 2 mmHg on the pressure dial at a time. The pressure that the patient could hold steady for 10 seconds with minimal superficial muscle activity is the one which was taken as baseline endurance capacity (10 repetitions of 10-second hold). The action was ensured to be pure nod with no head retraction and no head lifting. Subjects were told to perform head nodding action to gradually target and hold the 5 pressure levels for 10 seconds between 22 mmHg and 30 mmHg. The minimum satisfactory performance requirements were 26 mmHg. Each session of CCFT consisted of 3 sets, with each set having 10 repetitions. Sessions were performed for a total of 4 weeks with 4 days in a week under the supervision of the therapist. A two-minute rest was given between the sets.

## 3. Data Analysis

FHP was measured using a CVA in photographic analysis, functional status was measured using NDI scores, and pain intensity was measured using VAS scores. All statistical analyses were performed using the SPSS statistical software version 26 (SPSS Inc., Chicago, IL, USA). The Shapiro-Wilk test of normality was performed to assess the normal distribution of demographic data of all participants and the normal distribution of dependent variables (CVA, NDI, and VAS) data in both groups. Levene's test for equality of variances was performed to compare baseline values of dependent variables across both groups and revealed no significant difference CVA (*p* = 0.88), NDI (*p* = 0.16), and VAS (*p* = 0.41). Baseline values and values at the end of 4^th^ week were compared for all dependent variables. For the within-group comparison of CVA, a paired *t*-test was applied, and for NDI and VAS, the Wilcoxon signed-rank test was applied, because NDI data is considered an ordinal data and VAS data did not support normality. The ANOVA test was performed for the between-group analysis of mean differences of CVA at baseline and after 4 weeks of intervention. For between-group analysis of NDI and VAS, the Mann-Whitney *U* test was applied. Results in this study were considered significant if *p* < 0.05.

## 4. Results

**Figure 1:**
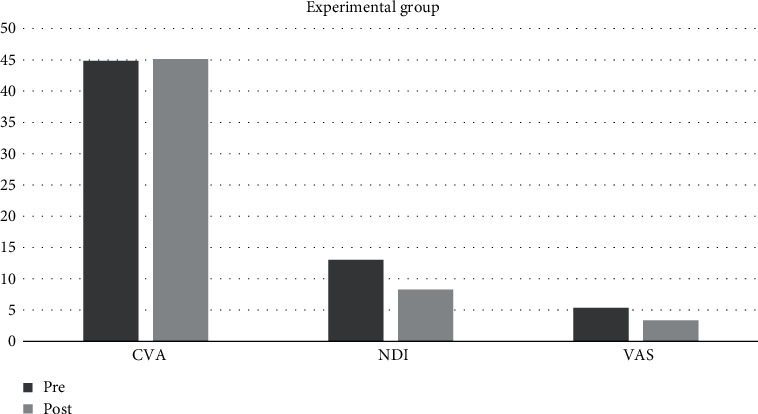
Graph depicting CVA, NDI, and VAS Pre and Post mean values in the experimental group.

**Figure 2:**
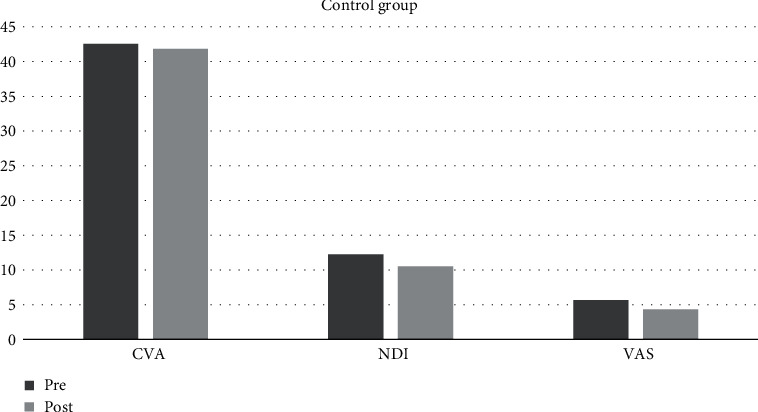
Graph depicting CVA, NDI, and VAS Pre and Post mean values in the control group.

The Shapiro-Wilk test of normality revealed a normal distribution of demographic data of all participants. CVA, NDI, and VAS mean values at baseline (Pre) and 4-week interval (Post) are presented in Table 2, Figure 1 and 2. The Shapiro-Wilk test of normality of mean values of dependent variables (CVA, NDI, and VAS) revealed normal distribution except for NDI Post mean value in the experimental group, VAS Pre value in the control group, and VAS Post value in the experimental group (Table 2).

### 4.1. Craniovertebral Angle (CVA)

The within-group analysis (paired sample test) revealed no significant improvement in CVA in both groups: experimental (*p* = 0.797) and control (*p* = 0.563) (Table 3). This means significant changes in CVA (i.e., CVA Pre-CVA Post) did not occur in both groups. Between-group (ANOVA) analysis of changes in CVA (i.e., CVA Pre-CVA Post) revealed no significant difference between the experimental group and the control group (*p* = 0.542) (Table 4).

### 4.2. NDI

The within-group analysis (Wilcoxon signed-rank) test revealed significant improvement in NDI in both groups: experimental (*p* = 0.001) and control (*p* = 0.036) (Table 3). This means NDI was improved significantly in both groups. Between-group analysis (Mann-Whitney *U* test) revealed that changes in NDI (i.e., NDI Pre-NDI Post) in the experimental group were significantly greater than changes in NDI in the control group (*p* = 0.019) (Table 4).

### 4.3. VAS

The within-group analysis (Wilcoxon signed-rank) test revealed significant improvement in VAS in both groups: experimental (*p* = 0.001) and control (*p* = 0.010) (Table 3). This means VAS was improved significantly in both groups. Between-group (Mann-Whitney *U* test) analysis of changes in VAS (VAS Pre-VAS Post) revealed no significant difference between the experimental group and the control group (*p* = 0.412) (Table 4).

## 5. Discussion

Results of the present study showed that pain and functional status were significantly improved in both the experimental group and the control group; however, there was no significant change in FHP in both groups. Also, there was no significant difference between both groups for FHP and neck pain. There was a significant improvement in functional status in the experimental group in comparison to the control group.

This is the first study, in our knowledge, where CCFT is used in the computer-using adolescents (age group 13 to 18 yrs.), having FHP and neck pain. Therefore, there is limited scope for comparison with other studies due to the lack of enough literature in this area. In patients with neck pain, DCF muscles' endurance capacity is lost [23]. These muscles have a significant role in maintaining cervical lordosis particularly in functional midranges of the cervical spine [24]; therefore, DCF training was chosen as a treatment for FHP in this study.

Our findings suggest that DCF training when given along postural education in the experimental group or postural education alone as given in the control group has no significant effect on FHP. Our findings are consistent with the results of Jull et al. [18] where the effectiveness of a 6-week low load craniocervical flexion exercise program in cervicogenic headache patients was studied through a randomized controlled trial. The result showed that there was a substantial decline in pain related to joint palpation and neck movement; however, the photographic measure of CVA representing FHP was unchanged [18]. Our results are also consistent with the findings of Kang, which reported significant improvement in neck muscle's endurance and ROM but no significant improvement was obtained in the CVA by performing pressure biofeedback-guided DCF muscles' training [25]. Grant et al. performed a single case study and reported no significant change in posture parameters (CVA) of the screen-based operator even though endurance of DCF increased and mechanosensitivity of articular, muscular, and neural structures reduced, after DCF and lower scapular muscle group's stabilization training of 4 weeks [26]. However, a study by Gupta et al. showed contrasting results than our findings and reported significant improvement in FHP as a result of DCF training [15].

A possible attempt has been made to explain the above findings. One of the primary reason could be that school pupil's head and neck posture is affected by multiple factors [27]. Computer furniture, anthropometric variations, reports of pain and visual factors, and potential harmful developmental effects occurring as a result of consistent postural stresses, all can affect school pupils' posture. Hence, just analyzing one factor in isolation and rectifying it would not prevent pupils from developing these musculoskeletal symptoms. Therefore, it is necessary to have a multidimensional approach if we want to have a significant and sustainable improvement in symptoms [27].

Another possible factor that could have influenced our results is that according to Janda from the viewpoint of muscle analysis, FHP is a result of the weakness of the DCF and dominance or even tightness of the sternocleidomastoid along with tightness of cervical extensor muscles [28]. There may be muscle imbalance around the cervical spine; therefore, activating a single muscle group in isolation may not be expected to be beneficial. Therefore, conclusions that could be drawn from our study are that DCF training in isolation may be ineffective in the treatment of FHP rather than being dismissive of any effectiveness of the exercise program. Addressing the muscle imbalance is important so that the optimal flexibility of those muscles that are tight could be achieved. Also, improving the strength of those muscles that are prone to weakness is necessary [28].

Apart from the above-stated explanation, another possible cause of our findings could be that static measure of FHP, i.e. CVA in the photographic analysis may not be an adequate outcome measure. Szeto in his study on comparison of computer workers with and without pain reported that computer users having neck pain drift into a more FHP when distracted [29]. This may indicate that muscles required to maintain the posture of the cervical spine have impaired or low level of endurance. It could also be argued that using photographs to measure spinal posture in an institution may not reflect ongoing posture. Also, the outcome of this study could have been influenced by several other less significant factors such as neck length, height, body built, genetic predisposition, or recreational activities of students.

The group receiving CCFT along with postural education showed greater statistical improvement in functional status (disability) in comparison to the group receiving only postural education. However, changes in FHP and neck pain were statistically similar in the group receiving both CCFT and postural training and group receiving postural education only. The possible explanation for the above findings could be that in our study, the total duration of DCF training was modified to once a day, 4 days in a week, for a total of 4 weeks, from the study performed by Jull et al. that included exercise protocol of twice a day, all days a week, for a total of 6 weeks. In our study, pupils performed every session in the supervision of a therapist; however, in the study of Jull et al., subjects performed exercises at home and were supervised by a therapist only once in a week [30]. This protocol was modified so that it suits pupils' schedules and school time table. Also, since it is better to perform exercises under the therapist's supervision than performing at home, therefore, each session was performed under the therapist's guidance. It could be possible that the 4-week duration of DCF training may not have been sufficient to produce adequate changes in DCF. Another significant outcome of this study will be the role of postural instructions in bringing significant improvement in functional status (disability) and pain in both groups. Deviation of posture from normal alignment causes imbalances and abnormal strains on the musculoskeletal system [20]. Based on this concept that postural imbalances or abnormalities cause injuries and pain, postural correction and education were used as a treatment approach for alleviating symptoms [20] and proved to be successful in this study.

### 5.1. The Relevance of the Study

Since the number of children using computers/laptops will increase in years to come, therefore, the risk for developing musculoskeletal disorders at a younger age will also increase. All this could increase sick leaves and early retirements. Education of correct body postures, ergonomic advice, and good work practices, when established in early life, significantly reduces the chances of developing musculoskeletal problems in later life.

### 5.2. Recommendation for Further Studies

Electromyography can be used in future studies to know simultaneous muscle activation occurring due to DCF training along with postural changes. Future studies are needed that addresses musculoskeletal imbalances (muscle shortening, etc.) also. Finally, instead of using a static photographic measure for FHP, a more dynamic outcome measure should be taken.

## 6. Conclusion

This study showed that DCF training and postural education of 4 weeks does not cause any significant improvement in FHP in adolescent pupils using computers regularly. But neck pain and functional status (perceived disability) improved significantly with DCF training and postural education either given alone or in combination with each other.

Significant improvement occurred in functional status when DCF training and postural education were given in combination with each other; however, in terms of reduction of neck pain, and FHP, there was no difference whether DCF training and postural education were given in combination or given alone. Therefore, this study cuncludes that 4 weeks of DCF training did not cause any significant improvement in FHP in adolescent pupils who use computers regularly.

## Figures and Tables

**Table 1:** Respondent's demographic data, *n* = 15 each group, mean ± SD, and *p* values for Shapiro-Wilk tests of normality.

	Experimental group	*p* value	Control group	*p* value
Age (years)	15.46 ± 1.88	0.07	15.93 ± 1.57	0.33
Height (cm)	165.2 ± 5.97	0.14	164.6 ± 6.55	0.16
Weight (kg)	48.8 ± 4.69	0.30	48.46 ± 3.50	0.99
BMI (kg/m^2^)	17.86 ± 0.84	0.57	17.90 ± 0.77	0.45

**Table 2:** CVA, NDI, and VAS, *n* = 15 each group, mean ± SD values at baseline (Pre) and after 4 weeks of intervention (Post). And *p* values for Shapiro-Wilk tests of normality.

	Experimental group	*p* value	Control group	*p* value
CVA Pre (degrees)	44.85 ± 7.54	0.48	42.55 ± 8.04	0.55
CVA Post (degrees)	45.13 ± 5.93	0.36	41.83 ± 8.33	0.08
NDI Pre (points)	13.00 ± 6.61	0.07	12.26 ± 5.29	0.38
NDI Post (points)	8.26 ± 5.67	0.02^∗^	10.53 ± 4.79	0.23
VAS Pre (cm)	5.33 ± 1.67	0.17	5.66 ± 1.91	0.02^∗^
VAS Post (cm)	3.33 ± 1.39	0.01^∗^	4.33 ± 1.58	0.18

^∗^Significant (*p* < 0.05).

**Table 3:** Within-group comparison for dependent variables, mean difference ± SD in both groups.

	Experimental group	SEM	*p* value	Control group	SEM	*p* value
CVA Post-CVA Pre	0.28 ±4.16	1.07	0.79	-0.71 ±4.70	1.21	0.56
NDI Post-NDI Pre	-4.73 ±3.49	0.90	0.00^∗^	-1.73 ±2.84	0.73	0.03^∗^
VAS Post-VAS Pre	-2.00 ±1.64	0.42	0.00^∗^	-1.33 ±1.67	0.43	0.01^∗^

^∗^Significant (*p* < 0.05); SEM = standard error of mean.

**Table 4:** Between-group comparison of dependent variables, mean difference ± SD.

	Experimental group	Control group	*p* value
CVA Post-CVA Pre	0.28 ±4.16	-0.71 ±4.70	0.54
NDI Post-NDI Pre	-4.73 ±3.49	-1.73 ±2.84	0.01^∗^
VAS Post-VAS Pre	-2.00 ±1.64	-1.33 ±1.67	0.41

^∗^Significant (*p* < 0.05).

## Data Availability

The data associated with the paper are not publicly available but are available from the corresponding author on reasonable request.
